# Tart Cherry Reduces Inflammation in Adipose Tissue of Zucker Fatty Rats and Cultured 3T3-L1 Adipocytes

**DOI:** 10.3390/nu10111576

**Published:** 2018-10-25

**Authors:** Shasika Jayarathne, April J. Stull, Alexandra Miranda, Shane Scoggin, Kate Claycombe-Larson, Jung Han Kim, Naima Moustaid-Moussa

**Affiliations:** 1 Department of Nutritional Sciences, Obesity Research Cluster, Texas Tech University, Lubbock, TX 79409, USA; shasika-har.udahawatte@ttu.edu (S.J.); alexandra.s.miranda@ttu.edu (A.M.); shane.scoggin@ttu.edu (S.S.); 2 Department of Human Ecology, University of Maryland Eastern Shore, Princess Anne, MD 21853, USA; ajstull@umes.edu; 3 USDA-ARS, Grand Forks Human Nutrition Research Center, Grand Forks, ND 58203, USA; Kate.Larson@ARS.USDA.GOV; 4 Department of Biomedical Sciences, Joan C. Edwards School of Medicine, Marshall University, Huntington, WV 25755, USA; kimj@marshall.edu; 5 Department of Plant and Soil Science, Texas Tech University, Lubbock, TX 79409, USA

**Keywords:** tart cherry, obesity, inflammation, adipose tissue

## Abstract

Obesity increases adipose tissue inflammation and secretion of pro-inflammatory adipokines, which have systemic effects on the organism’s health status. Our objective was to dissect mechanisms of anti-inflammatory effects of tart cherry (TC) in adipose tissue of Zucker fatty rats, and cultured 3T3-L1 adipocytes. Rats were fed either a control diet, or 4% TC powder diets for eight weeks. Body and epididymal fat pad weights were not significantly different between control and TC groups. However, rats fed the TC diet had significantly reduced adipose tissue inflammation (*p* < 0.05), as determined by reduced mRNA levels of pro-inflammatory markers including interleukin-6 (IL-6), tumor necrosis factor alpha (TNFα), interleukin-1beta (IL-1β), monocyte chemoattractant protein 1 (MCP-1), inducible nitric oxide synthase (iNOS), and CD-11b, and increased mRNA levels of type-1 arginase (Arg-1) anti-inflammatory marker. Consistent with these in vivo results, TC significantly decreased expression of IL-6 mRNA and protein levels in lipopolysaccharide (LPS) stimulated adipocytes compared to those stimulated with LPS, but no TC. Moreover, both in vivo (rat adipose tissue) and in vitro (3T3-L1 adipocytes), phosphorylation of p65-NF-κB subunit was significantly reduced by TC. Additionally, TC decreased mRNA expression of fatty acid synthase (FASN), and increased expression of peroxisome proliferator-activated receptor alpha (PPARα), master regulator of lipid oxidation, and anti-oxidant markers nuclear factor erythroid-derived 2-related factor (NRFs) in both models. In conclusion, our findings indicate that TC downregulates inflammation in part via the nuclear factor kappa B (NF-κB) pathway in adipose tissue. Thus, TC may serve as a potential intervention to reduce obesity-associated inflammation.

## 1. Introduction

Obesity is a disease affecting about 40.4% women, 35% men, and 17.0% adolescent and children in the United States [1]. Worldwide adult obesity reached over 650 million in 2016 [2]. Obesity is a well-known risk factor for other chronic diseases, such as type 2 diabetes (T2D), stroke, heart diseases, and cancer [3].

Obesity is a pro-inflammatory condition in which adipocytes enlarge due to excessive fat accumulation and increased secretion of pro-inflammatory molecules by adipose tissue [4]. Adipose tissue is important for energy homeostasis and it functions as an endocrine organ. More than 50 hormones and signaling molecules secreted by the adipose tissue, collectively called adipokines, play biological roles in an autocrine, paracrine, or systemic manner and affects numerous physiological processes regarding energy, glucose metabolism, and immunity [5]. Adipokines are either anti-inflammatory or pro-inflammatory [6]. Anti-inflammatory adipokines secreted from adipose tissue include interleukin 10 (IL-10), and adiponectin [7], and pro-inflammatory adipokines include tumor necrosis factor alpha (TNFα), interleukin 6 (IL-6), interleukin-1beta (IL-1β), and monocyte chemoattractant protein 1 (MCP-1) [7]. The disproportion between the secretion of pro- vs. anti-inflammatory adipokines increases macrophage infiltration into adipose tissue. Adipose tissue macrophages mainly fall into two categories: M1 pro-inflammatory and M2 anti-inflammatory. M1 macrophages increase the production of pro-inflammatory cytokines, such as tumor necrosis factor alpha (TNF-α), IL-6, and MCP-1 [8]. In contrast, M2 macrophages are involved in the increased secretion of anti-inflammatory cytokines, such as IL-10 and type-1 arginase (Arg-1) [9]. Therefore, increased number of M1 macrophages during obesity contribute to increased circulating levels of pro-inflammatory cytokines, which is further involved in the development of insulin resistance [10], where adipocytes and muscle cells reduce their responses to the normal function of insulin [11].

Dietary interventions using natural bioactive food compounds have arisen as favorable therapeutic approaches for obesity, inflammation, and metabolic disorders, with reduced adverse side effects [12,13,14]. Dietary supplementation with flavonoids exerts inflammation and body fat reducing effects [15,16,17]. Tart cherry (TC) (*Prunus cerasus*) contains the greatest concentrations of anthocyanin flavonoids among vegetables and fruits, including other cherries [18,19]. The total phenolic content of tart cherries (over 300 mg/100 g) is two to three times higher than that of sweet cherries [19,20]. Unlike flavonoid-containing green tea, TC has excellent palatability and familiarity to U.S. consumers due to its popularity in foods, such as pie, pastry fillings and juice products.

Most studies have shown that the consumption of anthocyanin-rich food reduces the development of chronic diseases, including diabetes, cardiovascular diseases, atherosclerosis, arthritis, and cancers [21,22,23,24]. In addition, many in vitro and in vivo studies have also documented the beneficial effects of anthocyanins on lipid metabolism, oxidative stress, inflammation, hyperglycemia, and visceral fat accumulation and insulin signaling [25,26,27,28]. However, many studies have used strawberry, blueberry, and sweet cherry anthocyanin or extracts to reduce such disease conditions, and few studies have documented the effects of TC anthocyanin in adipose tissue inflammation [27,29,30,31]. The objective of this study is to identify the potential anti-inflammatory activities of TC extract rich with anthocyanins in adipose tissue inflammation in obese Zucker fatty rats (OZFR) and cultured 3T3-L1 adipocytes. The OZFR were studied because they are an experimental model of metabolic syndrome [32] that resembles humans with metabolic syndrome. The 3T3-L1 adipocytes were used to further validate the in vivo results and to investigate possible major cellular mechanisms underlying TC regulation of adipose tissue inflammation. Our results discussed below showed that TC reduces adipose tissue inflammation in part via decreased nuclear factor kappa B (NF-κB) activation in both OZFR and cultured 3T3-L1 adipocytes. Additionally, we found fatty acid metabolism effects and anti-oxidant effects of TC in both models, which may indirectly downregulate inflammation.

## 2. Materials and Methods

### 2.1. Animal Studies

Seventeen-week-old male Zucker fatty rats (OZFR) were fed either a control (*n* = 11) or TC (*n* = 11) diet for 8 weeks. The cherry diet contained 4% cherry powder (Cherry Marketing Institute; TD.120586) by weight within a 2016 Tekland Global pellet diet, and the control diet (TD.120587) contained 4% extra carbohydrate by weight (dextrose:fructose, 1:1) to control for the additional carbohydrate provided by the cherry powder, thus yielding the two diets as isocaloric (70% carbohydrates, 20% protein, and 10% fat). The cherry diet was stored at −80 °C and the control diet was stored at 4 °C. The diets were provided fresh twice a week. After 8 weeks, body weight was measured, and rats were sacrificed by deep isoflurane followed by thoracotomy and cardiac puncture. Serum and epidydimal adipose tissue samples were collected and stored at −80 °C until further analyses. Serum MCP-1, IL-6 and IL-10, adiponectin, and leptin were measured using a 27-plex kit (RECYTMAG-65K | MILLIPLEX MAP Rat Cytokine/Chemokine, Millipore Sigma, Burlington, MA, USA), and serum cholesterol, triglyceride (TG), and glucose were analyzed on the Beckman Coulter DxC 600 analyzer (Brea, CA, USA). Serum insulin was analyzed using the Crystal Chem ELISA KIT (Elk Grove Village, IL, USA). The Institutional Animal Care and use committee of Pennington Biomedical Research Center (Baton Rouge, LA, USA) approved all the procedures (Protocol number 786). For the animal study, we used the freeze-dried powder from individually quick frozen (IQF) Montmorency tart cherries, which were prepared by VanDrunen Farms (Momence, IL, USA). The nutritional information was analyzed by VanDrunen Farms and its subsidiary FutureCeuticals, and further anthocyanin analysis was previously reported [33,34], as measured by liquid chromatography mass spectrometry (LC-MS). The total phenolics in TC powder is 10,323 ± 1468 µg/g of gallic acid equivalents and contains 482 ± 56 anthocyanin expressed as µg/g dry weight of cyanidin 3-glucoside equivalents [33]. Cyanidin 3-sophoroside (4.1 ± 0.8 µg/g), cyanidin 3-glucosylrutinoside (375.7 ± 55.1 µg/g), cyanidin 3-glucoside (7.1 ± 0.9 µg/g), and cyanidin 3-rutinoside (226.1 ± 44.2 µg/g) are the major anthocyanins present in TC powder [33].

### 2.2. Cell Culture

3T3-L1 mouse embryo fibroblasts were cultured in humidified atmosphere of 5% CO_2_, 95% air at 37 °C. The cells were maintained in Dulbecco’s Modified Eagle’s Medium (DMEM) (Thermo Fisher, Pittsburg, PA, USA) containing antibiotics 1% penicillin-streptomycin (PNS) (Thermo Fisher, Pittsburg, PA, USA) and 10% fetal bovine serum (FBS) (Atlas Biologicals, Fort Collins, CO, USA). The cells were differentiated in DMEM plus 0.5 mM 1-methyl-3-isobutylxanthine (MIX), 0.25 µM dexamethasone (DEX). Insulin (10 ng/mL; Sigma-Aldrich, St. Louis, MO, USA) was added to induce the lipid accumulation of cells. Media was changed every two days until maximum differentiation occurred.

To reveal the cytotoxic ability of TC on adipocytes, we next assessed the cell viability of 3T3-L1 cells using different concentrations (12 µL/mL, 36 µL/mL, 72 µL/mL) of TC. After 22 h, cells were removed from the incubator and 5 mg/mL of Thiazolyl Blue Tetrazolium Bromide (Sigma, St. Louis, MO, USA) was added and dissolved in cell culture media in each well (*n* = 3). Then, cells were incubated for 2 h. After the incubation period, 1 mL of dimethylthiazol diphenyltetrazolium bromide (MTT) stop solution (Fisher Scientific, Hampton, NH, USA) was added. Solution was mixed gently. Then, absorbance of the homogenized cell was measured using a citation 3 image reader (Winooski, VT, USA). Absorbance of the background at 690 nm was subtracted from the 570 nm measurement.

### 2.3. 3T3-L1 Cell Culture Treatments

After confluent, for the qRT-PCR and ELISA cytokine assay, 3T3-L1 adipocytes were treated 4 h with 12 µL/mL TC extract (juice) obtained from frozen TC (Cherry Marketing Institute) while control had normal media. Dose was chosen based on pilot experiments. Then, media was replaced with lipopolysaccharide (LPS—200 ng/mL, Sigma-Aldrich, St. Louis, MO, USA) to stimulate inflammation in the absence or presence of the TC extracts for 18 h. The chemical and anthocyanins profile of freeze-dried TC has been previously reported [33,35]. The TC extract from frozen TC contains 12,665 ± 1321 µg/g total phenolics and 533 ± 47 µg/g total anthocyanin. We have identified some of these anthocyanins, such as cyanidin 3-glucoside, cyanidin 3-rutinoside, and cyanidin using high performance liquid chromatography mass spectrometry (HPLC-MS) (Figure S1). The TC extract we used for cell culture studies contained 36 µg/mL total anthocyanins [36]. For western blot analyses, 3T3-L1 adipocytes were treated 21 h with 12 µL/mL TC extracts while the control had normal media. Then, media was replaced with 200 ng/mL LPS in the absence or presence of the TC extracts for 1 h to analyze the expression of phosphoproteins.

### 2.4. Anthocyanin Analysis of Frozen Tart Cherry Used in the Cell Culture Study (Liquid Chromatography Mass Spectrometry—LC-MS)

LC-MS was optimized with standards before running actual samples. Anthocyanin standards were purchased from Sigma Aldrich Company, St. Louis, MO, USA and consisted of Cyanidin Chloride (MW-322.70 g/mol), Keracyanin Chloride (Cyanidin-3-*O*-rutinoside chloride, MW-630.98 g/mol), Kuromanin Chloride (Cyanidin 3-*O*-glucoside chloride, MW-484.84 g/mol). (St. Louis, MO, USA). All the anthocyanin standards were dissolved in 80% methanol. Then, standards were further diluted to 100 µM using 0.2% Glacial Acetic Acid, 50% Acetonitrile and water.

Frozen TC were ground using a mortar and pestle. Then, TC juice was filtered using 0.22 µm filter unit. Tart cherry juice was freeze-dried using a vacuum desiccator. To extract anthocyanin, 1 g of freeze-dried tart cherry was dissolved with 10 mL methanol/water/acetic acid (85:15:0.5, *v*/*v*/*v*) and the tube was placed on a rocker at 4 °C overnight in the dark due to the sensitivity of anthocyanins to light. The sample was then vortexed, sonicated, and filtered through a 0.22 µm filter unit. Next, 1 mL of sample was dried for 3 h in the vacuum drier and 20 µL of anthocyanins were dissolved in 100 µL of 0.05% acetic acid and filtered using 3 K filter units (Millipore). Filtrate was re-diluted 10 times with 0.05% acetic acid for LC-MS analysis. The anthocyanin analyzed graph is included in Figure S1.

### 2.5. ELISA Cytokine Assay

Secreted pro-inflammatory (IL-6) adipokines in the cell culture media were measured using ELISA cytokine assay according to the instruction of the manufacturer (R&D systems, Minneapolis, MN, USA). Serial dilutions of standards of IL-6 were used to create a standard curve.

### 2.6. Gene Expression Analysis

RNA from 3T3-L1 adipocytes and epididymal adipose tissue from the rats was isolated using the Qiagen RNeasy Lipid Kit (Qiagen, Valencia, CA, USA) following the manufacturer’s protocol. Then, using iScript Reverse Transcription Supermix for qPCR (Bio-Rad Laboratories, Inc., Hercules, CA, USA), total RNA was reverse transcribed into cDNA. qPCR using iTag™ SYBER Green Supermix (Bio-Rad, Hercules, CA, USA) was made with cDNA to test target genes, including IL-6, TNF-α, MCP-1, IL-1β, NF-κB, Arg-1, Early growth response protein 2 (Egr-2), CD11-b, iNOS, toll-like receptors (TLRs), fatty acid synthase (FASN), peroxisome proliferator-activated receptor alpha (PPARα), and nuclear factor erythroid-derived 2-related factor (NRFs) with 18 s housekeeping gene (Sigma-Aldrich, St. Louis, MO, USA).

### 2.7. Protein Quantification and Western Blot Analysis

3T3-L1 adipocytes were collected and dissolved in RIPA mammalian protein extraction lysis buffer supplemented with protease phosphatase inhibitor (Pierce, Thermo Scientific, Carlsbad, CA, USA). Protein amount was quantified using a Bradford Assay Kit (Bio-Rad, Hercules, CA, USA) according to the manufacturer’s instructions. A total of 30 µg cell extract/well were loaded and, proteins were separated by any KD, Mini-Protean^®^ TGX Stain-Free™ gels (Biorad, Hercules, CA, USA). The gel with proteins was transferred to polyvinylidene fluoride (PVDF) (Bio-Rad, Hercules, CA, USA). The membranes were blocked with Pierce Blocking Buffer (Thermo Fisher Scientific, Waltham, MA, USA), then washed using Tris-buffered saline Tween-20 (TBST) solution, and phospho/total p-65, phospho/total mTOR, and phospho/total AMPK (dilution 1:1000) (Cell Signaling, Boston MA, USA) and Tubulin (dilution 1:2000) (Sigma, St. Louis, MO, USA) were used to probe overnight at 4 °C. After rinsing, membranes were incubated with secondary antibodies conjugated with horseradish peroxidase at dilutions of 1:10,000 for 1 h at room temperature. Protein bands were detected by LI-COR ODYSSEY CLx (Biosciences, Lincoln, NE USA) and quantified by Image Studio Version 4.0 to identify the difference between treatment groups.

### 2.8. Statistical Analysis

The means of in vivo data were analyzed using the student *t*-test and in vitro data were subjected to analysis of variance (ANOVA), followed by a Turkey’s post-hoc test. In vitro data are means of at least three independent experiments. All statistical analysis was conducted using GraphPad Prism Version 7.04 software. Data are presented as mean ± SEM (standard error of means), with statistical significance considered at *p* < 0.05.

## 3. Results

### 3.1. Tart Cherry Effects on Body Weight and Epidydimal Fat Pad Weight

**Figure 1:**
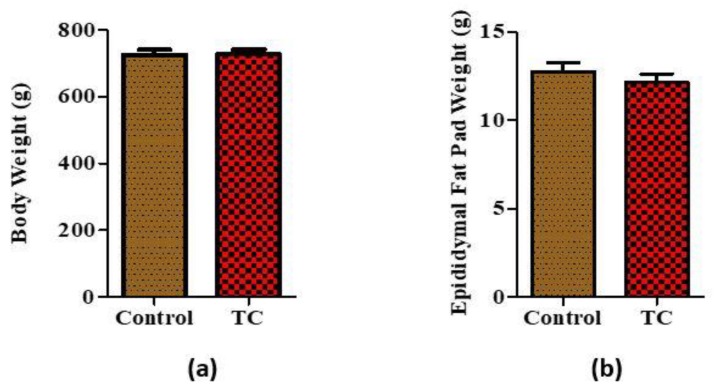
Body weight and epididymal fat pad weight measured at week 8 between control and tart cherry (TC) fed group. (**a**) Body weight measurements at week 8, (**b**) epididymal fat pad weights. (*n* = 11/group).

Overall, there were no significant differences between the average body weight and fat pad weight (*p* > 0.05) between rats fed with control compared to the TC group (Figure 1a,b).

### 3.2. Tart Cherry Effects in Serum Markers

There was no significant difference in serum metabolic markers at week 8 (*p* > 0.05) between the control and TC fed group (Table S1).

### 3.3. Tart Cherry Effects on Adipose Tissue Inflammation

**Figure 2:**
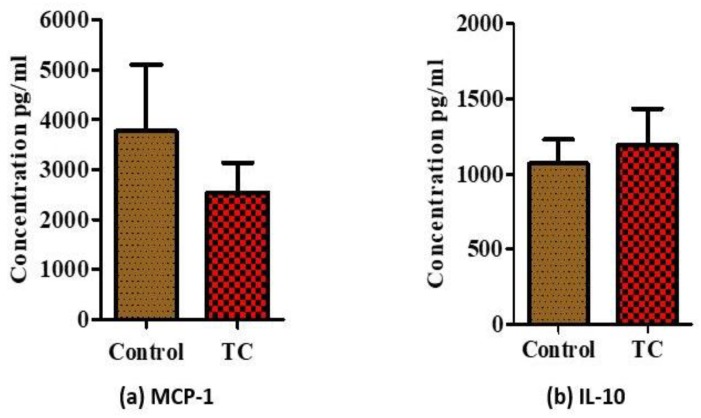
Changes of serum inflammatory markers. (**a**) Serum levels of proinflammatory monocyte chemotactic protein 1 (MCP-1), and (**b**) serum levels of anti-inflammatory interleukin 10 (IL-10) in Zucker fatty rats. (*n* = 11/group).

**Figure 3:**
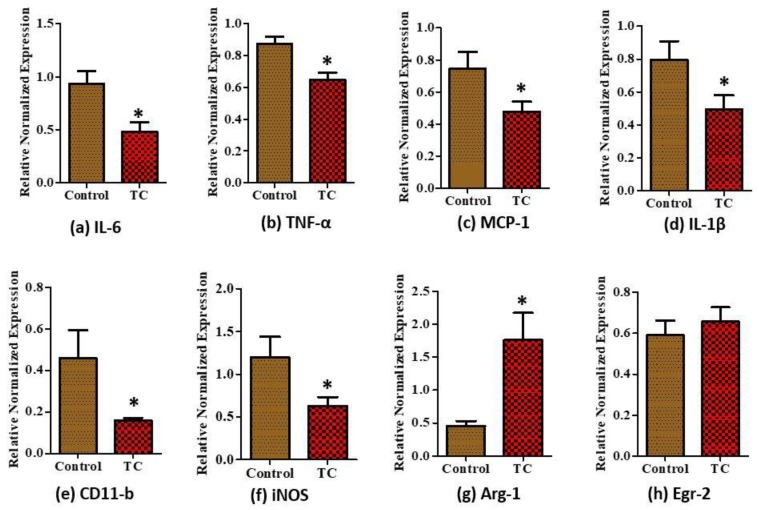
Relative normalized mRNA expression of inflammatory markers in epididymal fat of Zucker fatty rats. (**a**) Interleukin-6 (IL-6), (**b**) tumor necrosis factor alpha (TNF-α), (**c**) monocyte chemoattractant protein-1 (MCP-1), (**d**) interleukin-1beta (IL-1β) pro-inflammatory markers, (**e**) CD11-b M1 marker, (**f**) inducible nitric oxide (iNOS) M1 marker, (**g**) type 1 arginase (Arg-1) M2 marker, and (**h**) early growth response protein 2 (Egr-2) M2 marker in epididymal adipose tissue of Zucker fatty rats. * *p* < 0.05, control vs. tart cherry (TC), (*n* > 9/group).

There were no significant differences in serum pro- (MCP-1) vs. anti-inflammatory makers (IL-10) at week 8 (*p* > 0.05, Figure 2a,b). In the epidydimal fat, mRNA levels of pro-inflammatory IL-6, TNF-α, MCP-1, and IL-1β were significantly reduced in the TC group compared to the control group (Figure 3a–d, *p* < 0.05). Among the M1 markers, CD11b and iNOS showed significantly decreased (*p* < 0.05) expression in the TC group (Figure 3e,f). Moreover, Arg-1 M2 marker showed significantly increased expression in the TC group (*p* < 0.05) (Figure 3g). Egr-2 M2 marker had no difference in the TC group (Figure 3h).

**Figure 4:**
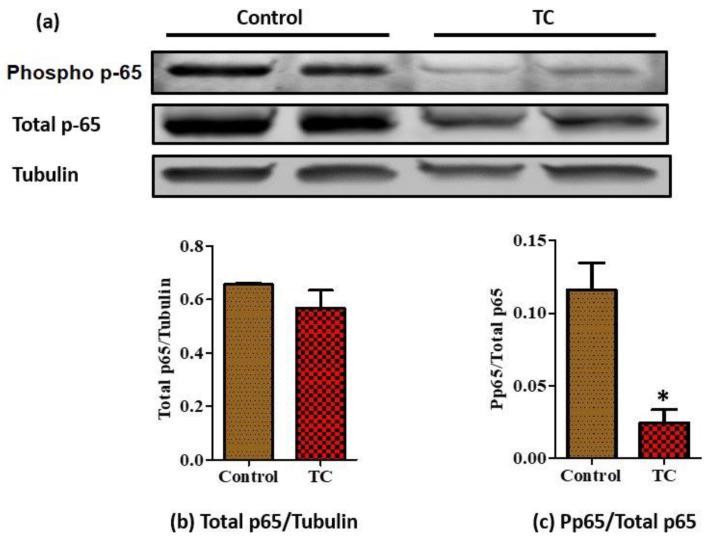
Expression levels of p-65 and tubulin proteins in epididymal fat of Zucker fatty rats detected by western blotting. (**a**) Phospho p-65, total p-65, and tubulin protein levels were examined using western blot. Tubulin was used as an internal loading control; (**b**) total p-65/Tubulin, (**c**) phospho p-65/Total p-65 (*n* = 8/group), two samples per group were used as representative images. * *p* < 0.05, control vs. tart cherry (TC).

Next, we analyzed changes of proteins involved in NF-κB pathways, one of the major pathways involved in adipose tissue inflammation. Interestingly, phosphorylation of p-65, the activator of NF-κB, was significantly reduced in epididymal adipose tissue of Zucker fatty rats by TC (Figure 4a; *p* < 0.05). Also, we measured IKBα, an inhibitor of the NF-κB pathway. However, there was no difference in IKBα protein levels between groups (data not shown). Additionally, we measured proteins involved in the mTOR and AMPK pathways related to inflammation and found no significant differences at protein levels in these pathways (Figure S2). Then, we evaluated the mRNA levels of TLRs to see the involvement of these receptors in the NF-κB pathway. However, TLRs did not show any significant reduction by TC (Figure S3).

### 3.4. Tart Cherry Effects on 3T3-L1 Cell Viability

**Figure 5:**
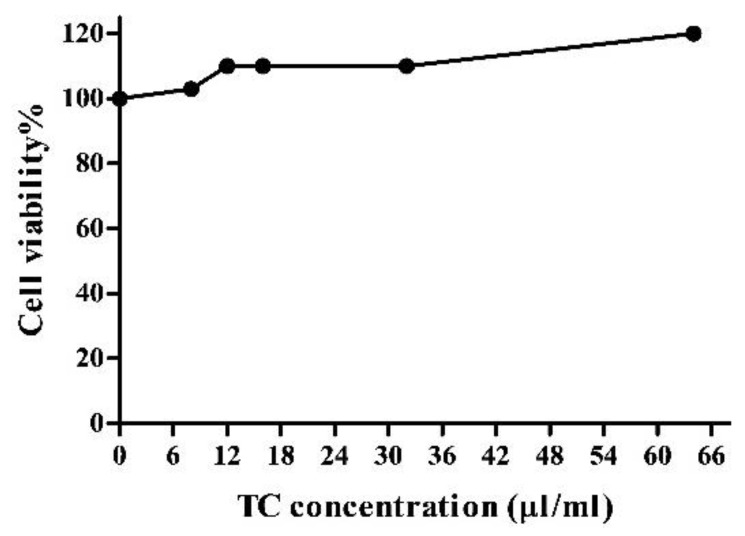
Cell viability of 3T3-L1 cells using different concentrations of tart cherry (TC) extract. MTT assay was performed to assess potential toxic effects of different concentrations of TC extracts. Tart cherry has no toxic effects on 3T3-L1 adipocytes, two independent experiments.

As shown in Figure 5, the cell viability increased with the TC concentration, showing that there were no cytotoxic effects of supplementation with TC extract in vitro.

### 3.5. Tart Cherry Effects on LPS-Induced Inflammation in 3T3-L1 Cells

Compared with the control, LPS treatment significantly increased IL-6 secretion in 3T3-L1 cells. Among the used TC concentrations, 3 µL/mL and 4 µL/mL did not show any significant reduction of IL-6 compared to LPS. However, 12, 18, and 24 µL/mL TC concentrations showed a significant reduction (*p* < 0.05) of IL-6 compared to the vehicle (Figure S4). Among those tested concentrations, we used 12 µL/mL TC to continue our cell culture experiments.

**Figure 6:**
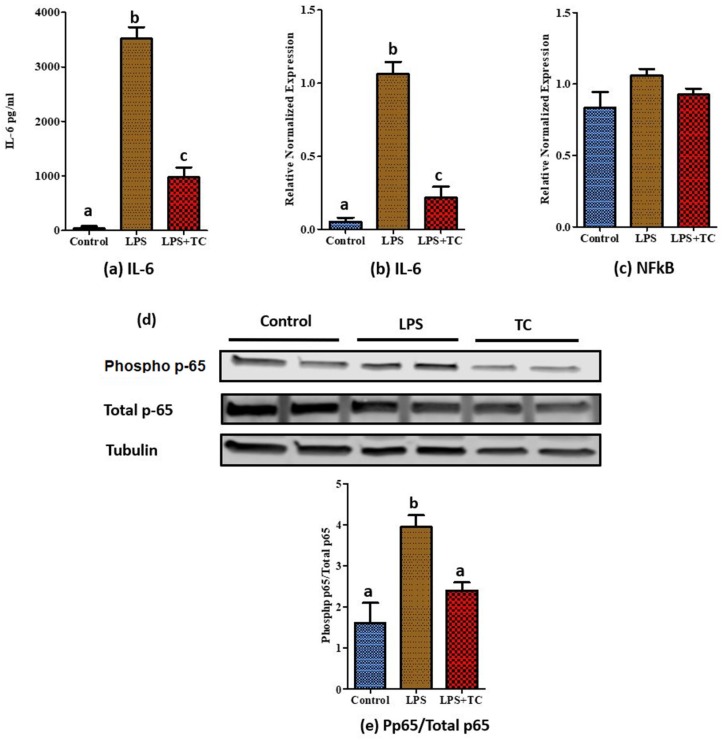
Inflammatory protein and relative normalized mRNA expression in tart cherry (TC) treated 3T3-L1 adipocytes. Cells were pre-treated for 4 h with 12 µL/mL TC extract, then media was changed to 200 ng/mL lipopolysaccharide (LPS) with or without TC while the control had normal media. (**a**) IL-6 secretion from 3T3-L1 adipocytes into media, (**b**) IL-6 mRNA expression, and (**c**) nuclear factor kappa B (NF-κB) mRNA expression, (**d**) phosphor-p-65, total p-65, and tubulin protein levels were examined using western blot. For measuring the expression of phospho protein, cells were pretreated for 21 h with 12 µL/mL TC extract, then media was changed to 200 ng/mL LPS with or without TC while the control had normal media. Tubulin was used as an internal loading control, (**e**) Phospho p-65/Total p-65. a, b, c different letters are significantly different, *p* < 0.05, four independent experiments.

Secretion of IL-6 proteins (Figure 6a) and the expression of IL-6 mRNA (Figure 6b), one of the major targets in the NF-κB pathway, was significantly reduced (*p* < 0.05) in TC treated 3T3-L1 adipocytes, as we observed in rat adipose tissue mRNA expression of NF-κB (Figure 6c), TNF-α, and MCP-1 (data not shown), which had no significant difference between the TC and vehicle groups. Furthermore, TC significantly reduced phospho-p65 (*p* < 0.05) in LPS treated 3T3-L1 adipocytes (Figure 6d,e). Similar to the observation in rat adipose tissue, we did not observe any differences in phospho-mTOR and phospho-AMPK protein levels in 3T3-L1 adipocytes treated with TC (Figure S5). Next, we analyzed the involvement of TLRs in the NF-κB pathway. There were no significant differences in the expression of TLR’s (TLR1, 3, 4, 6, and 7) between the TC and vehicle groups (Figure S6).

### 3.6. Tart Cherry Effects on Fatty Acid Metabolism

**Figure 7:**
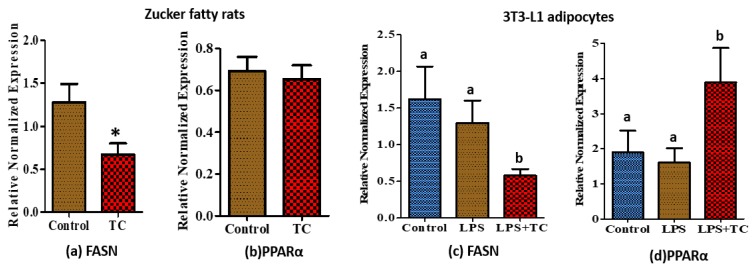
Relative normalized mRNA expression of fatty acid metabolic genes regulated by tart cherry (TC). (**a**) Expression of fatty acid synthase (FASN), (**b**) peroxisome proliferator-activated receptor alpha (PPARα) in epididymal adipose tissue of TC fed rats. * *p* < 0.05, control vs. tart cherry (TC), (*n* = 11/group), and (**c**) expression of FASN, (**d**) PPARα in TC treated 3T3-L1 adipocytes. a, b different letters are significantly different, *p* < 0.05, three independent experiments.

The mRNA expression of FASN was significantly reduced both in rat epididymal tissue and 3T3-L1 adipocytes by TC supplements (Figure 7a,c; *p* < 0.05). Moreover, PPARα expression was significantly higher in TC treated 3T3-L1 adipocytes (Figure 7d; *p* < 0.05), but no difference was observed in the epididymal adipose tissues of TC fed rats compared to the control (Figure 7b).

### 3.7. Tart Cherry Effects on Anti-Oxidant Activities

**Figure 8:**
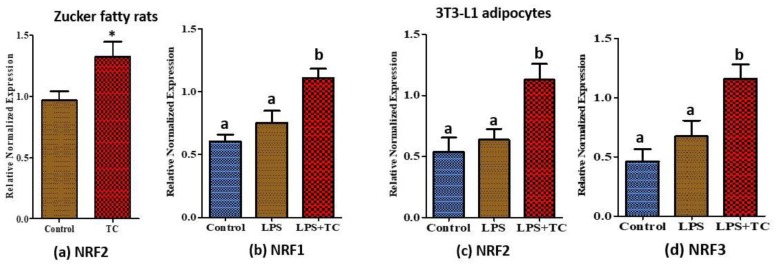
Relative normalized mRNA expression of anti-oxidant genes regulated by tart cherry (TC). (**a**) Expression of nuclear factor erythroid-derived 2-related factor-2 (NRF2) in epididymal adipose tissue of TC fed rats. * *p* < 0.05, control vs. tart cherry (TC), (*n* = 11/group) and the expression of (**b**) nuclear factor erythroid-derived 2-related factor-1 (NRF1), (**c**) NRF2, and (**d**) nuclear factor erythroid-derived 2-related factor-3 (NRF3) in TC treated 3T3-L1 adipocytes. a, b different letters are significantly different, *p* < 0.05, three independent experiments.

It has been well documented that anthocyanins possess anti-oxidant activity [37]. In addition to direct anti-inflammatory activities, we found anti-oxidant activities of TC in rat epididymal tissues and in 3T3-L1 adipocytes. For example, we tested the expression levels of NRFs 1, 2, and 3 to see whether TC can decrease the level of reactive oxygen species (ROS) in adipose tissue and adipocytes. Expression levels of nuclear factor erythroid-derived 2-related factor-2 (NRF2) were significantly higher in TC fed rats (Figure 8a; *p* < 0.05) compared to control, while the expression levels of all the tested NRF genes significantly increased with TC compared to the vehicle in 3T3-L1 adipocytes (Figure 8b–d; *p* < 0.05).

## 4. Discussion

Several studies have demonstrated that incorporating bioactive food components in the diet exerts multiple beneficial health effects, including decreased inflammation, obesity, and other metabolic disorders [38,39,40,41,42,43,44]. Recently, TC extract/anthocyanin has been well studied for its role of reducing symptoms of muscle damage/pain [45,46,47], muscle recovery and exercise performance [48,49], oxidative stress [50,51], and its beneficial effects on sleep [52,53]. However, limited studies have documented the positive effects of TC on inflammatory and metabolic effects [54,55,56]. Specifically, very few studies discussed the anti-inflammatory mechanisms of TC on adipose tissue or 3T3-L1 adipocytes [31,36]. In this study, we used both in vivo and in vitro models to investigate the effects of TC in adipose tissue inflammation. Here, we reported that TC decreased adipose tissue inflammation in Zucker fatty rats by reducing the expression of pro-inflammatory markers and increasing the expression of anti-inflammatory markers. The study showed cellular anti-inflammatory mechanisms regulated by TC in 3T3-L1 adipocytes, which are similar to findings in the obese Zucker rat model. Moreover, we showed anti-oxidant activity of TC in both tissue and cellular levels, which may be indirectly involved in reducing adipose tissue inflammation.

Increased adipose tissue mass during obesity increases the circulation levels of TNFα and other adipokines [57,58]. The levels of TNFα increase proportionally to adiposity and insulin resistance. IL-6 is one of the major inflammatory mediators, and its expression is inducible by other inflammatory lymphokines, such as IL-1 [59]. Like TNFα, the levels of IL-6 in plasma increase with fat mass and obesity. In this study, we observed reduced mRNA expression of TNFα, IL-6, and IL-1β in epididymal fat, which is consistent with previously published animal studies reporting TC effects in retroperitoneal fat [31]. Leptin is well known to regulate food intake and body weight; but also exhibits pro-inflammatory activities by up-regulating pro-inflammatory cytokines, such as TNFα, IL-12, and IL-6 [60]. However, in our current study, we did not identify any changes in fat pad weight or leptin levels with diet, suggesting that decreased inflammation in adipose tissue is independent from the leptin levels. This may be, in part, due to the animal model used, as the Zucker rats lack leptin receptors and are hyperleptinemic. Moreover, decreased IL-6 mRNA expression and IL-6 protein secretion were observed in the LPS stimulated 3T3-L1 adipocytes treated with TC. These results are consistent with a previously reported reduction of LPS induced IL6 in adipose stem cells by tart cherry anthocyanins [36].

Besides adipose tissue, TNFα can also be produced by adipose macrophages. Adipose tissue has classically activated M1 and alternatively activated M2 macrophages. Pro-inflammatory TNFα, IL-6, and MCP-1 can be secreted by M1 macrophages and anti-inflammatory IL-10, Arg-1, CD206, and Egr-2 by M2 macrophages [61]. Herein, we observed significantly reduced expression of macrophage derived M1 inflammatory markers, such as CD-11b, MCP-1, iNOS, and TNFα, and increased expression of Arg-1 M2 anti-inflammatory markers by TC in epididymal adipose tissue. These data show that anti-inflammatory mechanisms in adipose tissue may also be due to the changes in M1 and M2 macrophages.

NF-κB is a major inflammatory transcription factor, which regulates many downstream genes associated with inflammation, such as IL-6, MCP-1, and TNFα [61]. To validate the contribution of the NF-κB pathway in adipose tissue inflammation, we evaluated protein levels of p65, the NF-κB subunit. Surprisingly, phosphorylation of p65 was significantly reduced in epidydimal adipose tissue of the TC fed group. This may suggest that the reduced expression of TNFα and IL-6 may be partially caused by downregulation of NF-κB activity in adipose tissue. This agrees with findings by Seymour et al. showing reduced rat retroperitoneal fat NF-κB activity by TC [31]. Furthermore, it has been reported that TC supplementation increased mRNA levels of PPARα in retroperitoneal fat [31], a major transcription factor in fatty acid oxidation, which may also affect the genes involved in inflammation. Activation of PPRAα increases the activation of IkBα, an inhibitor of NF-κB [31]. In our study, we did not see any differences in mRNA levels of PPARα in the epididymal adipose tissue of rats. However, the mRNA levels of PPARα were significantly increased by TC treated 3T3-L1 adipocytes. Expression of fatty acid synthase (FASN), a key enzyme in de novo lipogenesis, correlates with increased adiposity [62], and is over expressed in adipocytes of obese rats [63]. It has been previously reported that rats fed with chokeberry extract exhibited lower mRNA levels of FASN in epididymal adipose tissue [10]. Moreover, anthocyanins inhibit lipid accumulation by reducing the gene expression levels of FASN of 3T3-L1 pre-adipocytes during adipocyte differentiation [64]. Similar to these findings, we also observed reduced mRNA expression of FASN in both rat epididymal adipose tissue and 3T3-adipocytes treated with TC.

Moreover, NF-κB can be activated by LPS. Upon exposure to inflammatory LPS stimuli, nuclear translocation of p65, the NF-κB subunit, regulates the expression of genes involved in inflammation [26]. Previous studies have reported the inhibition of the LPS induced NF-κB pathway using anthocyanins and anthocyanins-rich red raspberries, in cell models, such as BV2 microglial cells and raw 264.7 macrophages [26,65]. Here, we demonstrated reduced phosphorylation of p65 by TC in adipocytes. This suggests that similar mechanisms may be involved in anti-inflammatory responses to TC in both immune cells and adipocytes.

It is well known that dietary polyphenol possesses anti-oxidant activities [23]. Here, we evaluated the effects of TC on adipose tissue and 3T3-L1 cells because anti-oxidant activity may indirectly involve reducing inflammation. NRF2 is a master regulator of antioxidant responses and modulates the expression of many genes in response to anthocyanins [23]. In one study, NRF2 expression was increased and NF-κB activity was decreased in rats fed with the anthocyanin fraction of purple sweet potato [66]. Another study conducted in mouse macrophages showed that NRF2 binds to the promoters of pro-inflammatory cytokines, such as IL-6, and inhibits the recruitment of RNA polymerase II [67]. Moreover, knockdown of NRF2 significantly induces the transcription activity of NF-κB in response to LPS [68]. Apart from that, increased NRF2-dependent downstream heme oxygenase-1 (HO-1) expression inhibits NF-κB activity [57]. nuclear factor erythroid-derived 2-related factor-1 (NRF1), NRF2, and nuclear factor erythroid-derived 2-related factor-3 (NRF3) belong to the same regulatory protein family called the Cap-N-Collar family. We observed significant increases in expression levels of all tested NRF 1/2/3 in 3T3-L1 adipocytes treated with TC, and NRF2 in TC fed OZFR. These data further strengthen our main hypothesis of the anti-inflammatory activity of TC in adipose tissue, and further support the results of increased cell viability using higher doses of TC in cells, as shown in Figure 5. Indeed, the high anti-oxidant ability of various compounds may inhibit reactive oxygen forming enzymes that are harmful to cells [23].

TLRs are important mediators in innate immunity and their responses to attack pathogens have been well studied [69]. TLRs have an ability to identify molecules produced endogenously due to cell damage [69]. TLRs can be stimulated by LPS [70]. We observed that TLRs (TLR1, 3, 4, 6, and 7) are activated by LPS, but no significant inhibition by TC treatment (Figure S6). Consistent with cell culture data, our in vivo results also did not show any significant changes in TLRs between the TC and control group (Figure S3). In contrast to our findings, a study published by Liu et al. showed the inhibition of TLR4 activity by blueberry anthocyanins in the heart tissues of rats [71]. However, no studies have reported the inhibition of TLRs by anthocyanin rich fruit extract in 3T3-L1 cells or other cell culture models. Therefore, our findings may suggest that TC may act on the NF-κB pathway through different receptors, such as T-cell receptors and B cell receptors, but not through the TLRs in adipocytes. Moreover, we found no differences in the protein levels of mTOR and AMPK both in TC treated rat epididymal fat and 3T3-L1 adipocytes (Figures S2 and S5), suggesting that the main targeted pathway by TC may be the NF-κB and not the mTOR or AMPK pathways in adipose tissue to reduce inflammation. Additional large-scale RNA sequencing or proteomic studies may help identify additional mechanisms by which TC regulates adipose tissue metabolism and inflammation.

We used both in vivo and in vitro models to investigate major possible pathways mediating TC anti-inflammatory effects in adipose tissue. There are a few limitations in our study. First, we did not measure adipocyte area and infiltration of macrophages into adipose tissue of the control and TC groups. Next, we did use whole TC for our study. It would be worthwhile also to compare effects of isolated individual and combined anthocyanins from TC against whole extract to determine whether the anti-inflammatory effects are from anthocyanin or from whole TC extract as a synergistic effect due to other compounds in TC.

## 5. Conclusions

**Figure 9:**
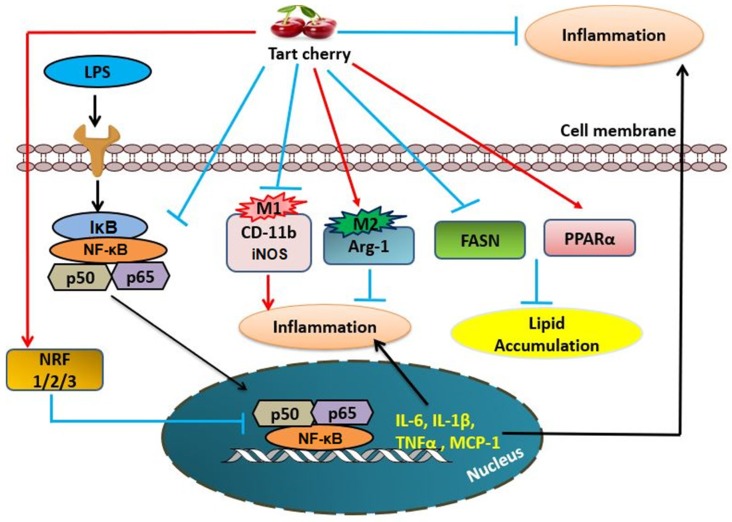
Proposed model for inhibition of adipose tissue inflammation by tart cherry. Tart cherry inhibits the NF-κB pathway and production of Pro-inflammatory markers in adipose tissue, namely CD-11b (M1 macrophage marker), inducible nitric oxide synthase (iNOS) (M1), tumor necrosis factor alpha (TNFα), monocyte chemoattractant protein 1 (MCP-1), interleukin-6 (IL-6), and interleukin-1beta (IL-1β) and increases the production of type-1 arginase (Arg-1) anti-inflammatory M2 marker. Moreover, tart cherry reduces the expression of fatty acid synthase (FASN) and increases the fatty acid beta oxidation (PPARα) and the expression of anti-oxidant markers, nuclear factor erythroid-derived 2-related factor-1 (NRF1), nuclear factor erythroid-derived 2-related factor-2 (NRF2), and nuclear factor erythroid-derived 2-related factor-3 (NRF3), which indirectly inhibit the inflammatory NF-κB pathway in adipose tissue.

In conclusion, our findings herein demonstrated that dietary TC supplementation may support metabolically healthy obesity through significantly reduced inflammation in adipose tissue, mainly through the NF-κB pathway. Additionally, our findings provide evidence that TC supplementation reduces fatty acid synthesis, changes the pro-inflammatory M1 macrophages, and increases the anti-oxidant activities in adipocytes/adipose tissue, which may indirectly help reduce adipose tissue inflammation (Figure 9). However, further studies are needed to investigate other possible pathways and cellular mechanisms regulated by TC in adipose tissue using both animal and cell models. It is also important to translate these findings clinically using human subjects.

## Supplementary Materials

The following are available online at http://www.mdpi.com/2072-6643/10/11/1576/s1, Figure S1: Anthocyanin analysis of tart cherry extract (used in cell culture) by high performance liquid chromatography mass spectrometry (HPCL-MS), Figure S2: Expression of phospho-mTOR and phospho-AMPK in epididymal adipose tissue of Zucker fatty rats, Figure S3: Relative normalized mRNA expression of toll like receptor (TLRs) in rat epididymal adipose tissue, Figure S4: Dose-dependent reduction of IL-6 secretion by 3T3-L1 cells by tart cherry extracts, Figure S5: Expression of phospho-mTOR and phospho-AMPK in 3T3-L1 adipocytes, Figure S6: Relative normalized mRNA expression of toll like receptors (TLRs) in 3T3-L1 adipocytes, Table S1: Effects of tart cherry on hormone and metabolic parameters in Zucker fatty rats.
