# Supplementary material for: Tart Cherry Reduces Inflammation in Adipose Tissue of Zucker Fatty Rats and Cultured 3T3-L1 Adipocytes
Source: Nutrients. 2018 Oct 25;10(11):1576. doi: 10.3390/nu10111576 (PMC6266132; doi:10.3390/nu10111576)
Supplement: Supplementary file 1 [file nutrients-10-01576-s001.pdf]

Standards	Retention time (min)	Molecular ion [m/z]	MS/MS fragmentation ions [m/z]
Cyanidin	20.82	287.10	213.07
C3R	15.42	595.11	287.06
C3G	14.87	449.22	287

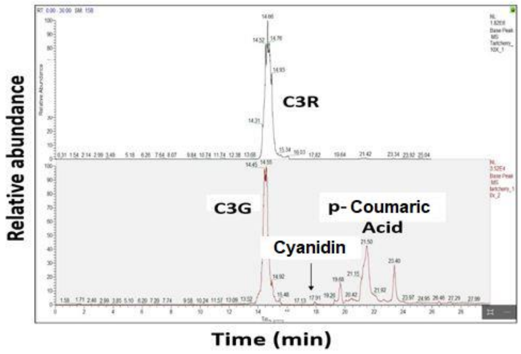

Figure S1. Anthocyanin analysis of tart cherry extract (used in cell culture) by high performance liquid chromatography mass spectrometry (HPLC-MS). Cyanidin-3-rutinoside (C3R) and Cyanidin-3-glucoside showed (C3G) high abundance in tart cherry.

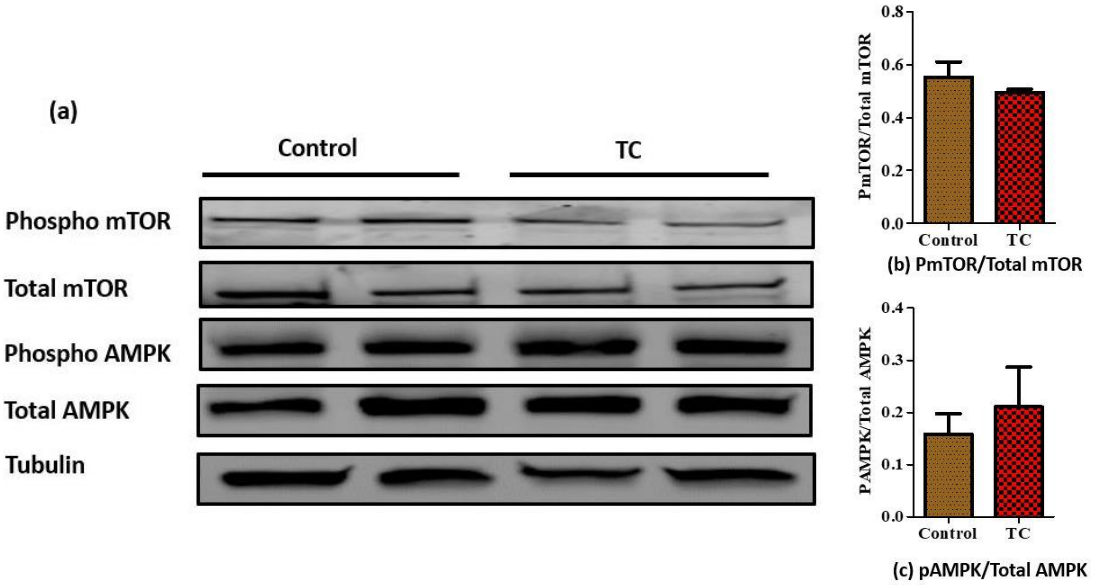

Figure S2. Expression of phospho-mTOR and phospho-AMPK in epididymal adipose tissue of Zucker fatty rats detected by western blot. (**a**) Phospho-mTOR, total mTOR, phospho-AMPK, total AMPK, and tubulin protein levels were examined using western blot. Tubulin was used as an internal loading control; (**b**) Phospho-mTOR/Total mTOR, (**c**) Phospho-AMPK/Total AMPK. $p > 0.05$, ($n = 8$/group).

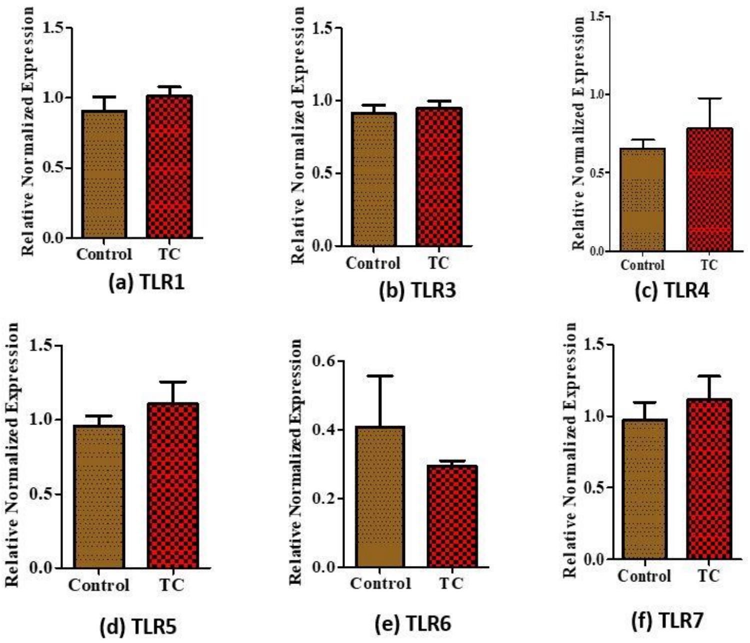

Figure S3. Relative normalized mRNA expression of toll like receptor (TLRs) in rat epididymal adipose tissue. None of the TLRs shown are reduced by tart cherry. $p > 0.05$, ($n = 11$/group).

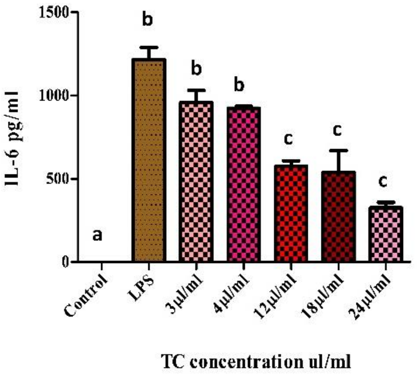

Figure S4. Dose-dependent reduction of IL-6 secretion by 3T3-L1 cells by tart cherry (TC) extracts. Lipopolysaccharide (LPS) induced IL-6 secretion from 3T3-L1 adipocytes was dose-dependently reduced by increasing concentrations of tart cherry extracts with 12 μL/mL achieving maximal effective dose. a, b, c different letters are significantly different, $p < 0.05$, three independent experiments.

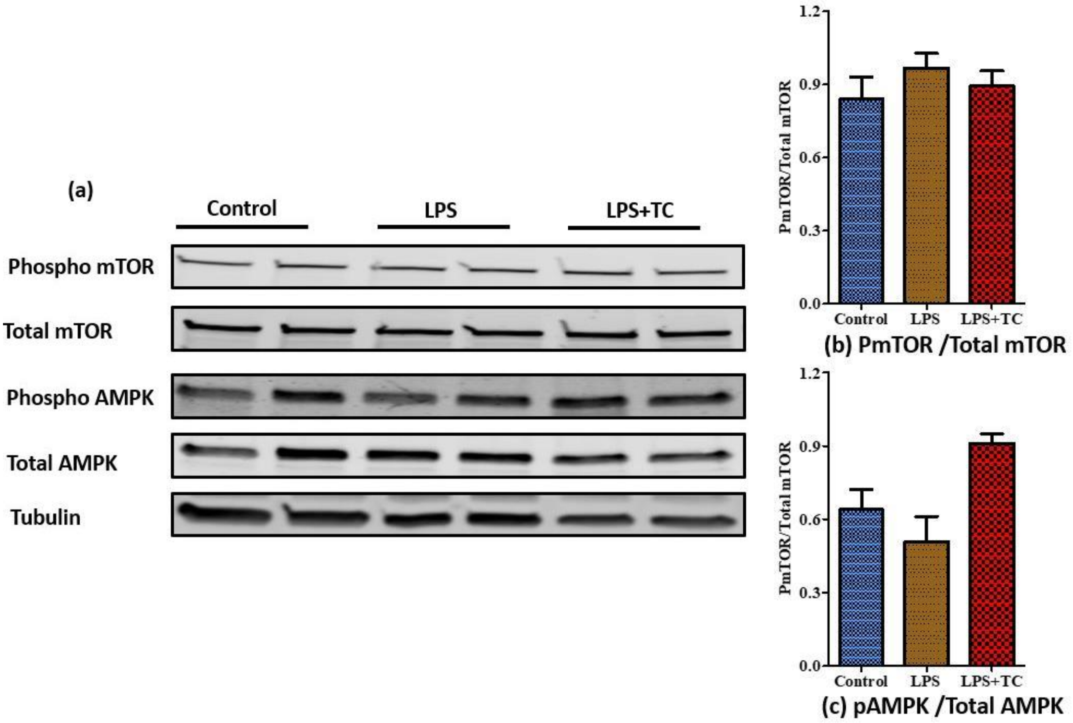

Figure S5. Expression levels of mTOR, AMPK and tubulin in 3T3-L1 adipocytes detected by western blot. (**a**) Phospho-mTOR, total mTOR, phospho-AMPK, total AMPK, and tubulin protein levels were examined using western blot. Tubulin was used as an internal loading control; (**b**) Phospho-mTOR/Total mTOR, (**c**) Phospho-AMPK/Total AMPK. $p > 0.05$, three independent experiments.

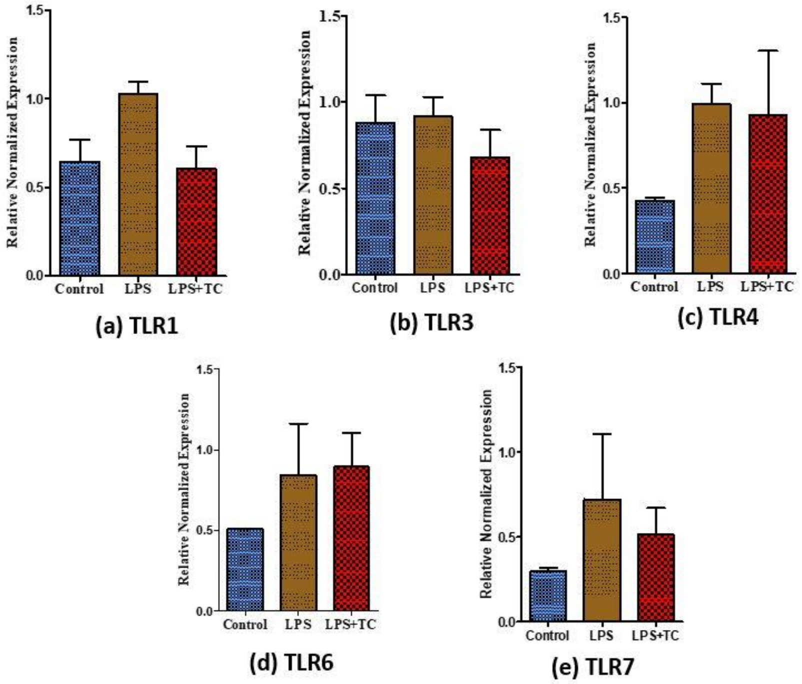

Figure S6. Relative normalized mRNA expression of toll like receptors (TLRs) in 3T3-L1 adipocytes. None of the TLRs tested show significant reduction by tart cherry treatment in 3T3-L1 adipocytes. $p > 0.05$, three independent experiments.

Table S1. Effects of tart cherry on hormone and metabolic parameters in Zucker fatty rats.

Parameter	Control	Tart cherry (TC)
Cholesterol (mg/dL)	211.2 ± 24.26	189.4 ± 18.72
Triglyceride (mg/dL)	576.3 ± 113.3	500.1 ± 66.78
Glucose (mg/dL)	158.8 ± 5.77	154.4 ± 6.66
Insulin (pg/mL)	4804 ± 475.6	5266 ± 510.9
Adiponectin (ng/mL)	3245 ± 163.1	3059 ± 209.3
Leptin	AD	AD

None of these values are significant. Values are expressed as the mean ± SEM (n = 11). Control, normal diet; TC, 4% TC diet (n = 11/group) at week 8. AD: above detection.
